# Benchmark of 16S rRNA gene amplicon sequencing using Japanese gut microbiome data from the V1–V2 and V3–V4 primer sets

**DOI:** 10.1186/s12864-021-07746-4

**Published:** 2021-07-10

**Authors:** Shoichiro Kameoka, Daisuke Motooka, Satoshi Watanabe, Ryuichi Kubo, Nicolas Jung, Yuki Midorikawa, Natsuko O. Shinozaki, Yu Sawai, Aya K. Takeda, Shota Nakamura

**Affiliations:** 1 Department of Infection Metagenomics, Genome Information Research Center, Research Institute for Microbial Diseases, Osaka University, Suita, Osaka Japan; 2 Cykinso, Inc. Shibuya, Tokyo, Japan; 3 Next-Generation Sequencing Core Facility, Genome Information Research Center, Research Institute for Microbial Diseases, Osaka University, Suita, Osaka Japan; 4 Integrated Frontier Research for Medical Science Division, Institute for Open and Transdisciplinary Research Initiatives, Osaka University, Suita, Osaka Japan; 5 Laboratory of Pathogen Detection and Identification, International Research Center for Infectious Diseases, Research Institute for Microbial Diseases, Osaka University, Suita, Osaka Japan

**Keywords:** Microbiota, 16S rRNA, Next-generation sequencing

## Abstract

**Background:**

16S rRNA gene amplicon sequencing (16S analysis) is widely used to analyze microbiota with next-generation sequencing technologies. Here, we compared fecal 16S analysis data from 192 Japanese volunteers using the modified V1–V2 (V12) and the standard V3–V4 primer (V34) sets to optimize the gut microbiota analysis protocol.

**Results:**

QIIME1 and QIIME2 analysis revealed a higher number of unclassified representative sequences in the V34 data than in the V12 data. The comparison of bacterial composition demonstrated that at the phylum level, Actinobacteria and Verrucomicrobia were detected at higher levels with V34 than with V12. Among these phyla, we observed higher relative compositions of *Bifidobacterium* and *Akkermansia* with V34. To estimate the actual abundance, we performed quantitative real-time polymerase chain reaction (qPCR) assays for *Akkermansia* and *Bifidobacterium*. We found that the abundance of *Akkermansia* as detected by qPCR was close to that in V12 data, but was markedly lower than that in V34 data. The abundance of *Bifidobacterium* detected by qPCR was higher than that in V12 and V34 data.

**Conclusions:**

These results indicate that the bacterial composition derived from the V34 region might differ from the actual abundance for specific gut bacteria. We conclude that the use of the modified V12 primer set is more desirable in the 16S analysis of the Japanese gut microbiota.

**Supplementary Information:**

The online version contains supplementary material available at 10.1186/s12864-021-07746-4.

## Background

The 16S rRNA gene (16S) is conserved in most bacteria and archaea and is approximately 1,500 bp long with nine different hypervariable regions (V1–V9). Amplicon sequencing targeting the 16S rRNA gene (16S analysis) is widely used to analyze microbiota using next-generation sequencing (NGS) technologies [1, 2]. Because of the limitation of short-read NGS technologies, various universal primers targeting the partial sequences in hypervariable regions (e.g. V1–V2, V1–V3, V3–V4, V4, etc.) have been developed for microbiome analysis. Furthermore, many experimental and analytical variations in the 16S analysis protocol have been reported [3, 4]. Currently, there is no gold standard method, and the standardization of various applications is an important issue in metagenomic analysis. For example, the DNA extraction process is a well-known influencing factor for metagenomic analysis. Careful comparisons between the bead-beating and enzymatic lysis methods have indicated how this extraction process affects the metagenomic data [5–7].

One of the most controversial issues in 16S analysis protocol variations is the selection of the hypervariable region(s) to target. Historically, the V1–V2 (V12) region has been employed in many reports of gut microbiota using past NGS technologies, for example, 454 pyrosequencing [8–10]. As the official Illumina protocol adopted the V3–V4 (V34) region, these two regions are widely used in gut microbiota studies [11–18]. Claesson et al. showed that the V34 primer-pair combination causes amplification artifacts and has a deviating composition compared to other regions, including V12 [19]. In contrast, Chen et al. reported that V34 is more suitable for gut microbiota analysis than V12 because it has a higher potential to detect the order Bifidobacteriales [20]. However, Kim et al. have developed a new version of the V1 forward primer (27Fmod) with improved *Bifidobacterium*-detection compared to that of the primer used by Chen et al. (27F-YM) [21].

This problem does not only exist for the gut microbiota. Comparative studies of environmental water reported that the V34 or V4 primer sets are optimal [22, 23]. A similar conclusion, the V34 region is more suitable than V12, was reported for human vaginal microbiome analysis [24]. Meanwhile, a comparative study of the human oral microbiome reported that V1–V3 is more suitable than V34 [25]. As shown in these examples, the choice of optimal variable regions might vary depending on the analysis target, the specificity of the primers, the GC contents in the selected region, and the bacterial compositions of different samples.

Data processing tools are another factor that impacts the interpretation of the microbiome. QIIME (Quantitative Insights Into Microbial Ecology), one of the most popular bioinformatics tools for 16S analysis, comprises version 1 (QIIME1: qI) released in 2011 and version 2 (QIIME2: qII) released in 2018 [26, 27]. qII has been completely redesigned as an algorithm for clustering amplicon sequence variants (ASVs) from operational taxonomic units (OTUs) in the previous version and is reported to allow for more accurate clustering of ASVs. However, in some cases, qI is still being utilized to compare the vast amount of data that have been produced in the past.

In this study, we focused on selecting the hypervariable regions of 16S rRNA for human gut microbiome analysis. To our knowledge, there have been no reports comparing the gut microbiota between the V12 region with 27Fmod and V34 region. To identify which primer set that is better suited for analyzing the Japanese gut microbiome, we compared the intestinal microbiome data from 192 Japanese volunteers using both primer sets, V12 (27Fmod) and V34. In addition, to evaluate those data with the previously published studies, a comparison between QIIME versions qI and qII was also conducted.

## Participants & methods

### Participants

A total of 192 healthy Japanese volunteers was randomly selected from the 1,644 samples in the Mykinso library recruited between July 2015 and August 2016. The age and gender distributions of the selected participants are shown in Table 1S.

### Fecal sampling, DNA extraction, and sequencing

We performed the V1–V2 region sequencing as reported in Watanabe et al. [28]. We collected fecal samples using brush-type collection kits containing guanidine thiocyanate solution (Techno Suruga Laboratory, Shizuoka, JPN), transported at ambient temperature, and stored at 4 °C. DNA was extracted from the fecal samples using the DNeasy PowerSoil Kit (QIAGEN, Hilden, DEU) according to the manufacturer’s protocol. The amplicons of V12 were prepared using the forward primer (16S_27Fmod: TCG TCG GCA GCG TCA GAT GTG TAT AAG AGA CAG AGR GTT TGA TYM TGG CTC AG) and the reverse primer (16S_338R: GTC TCG TGG GCT CGG AGA TGT GTA TAA GAG ACA GTG CTG CCT CCC GTA GGA GT). The amplicons of V34 were prepared using the forward primer (16S_341F: TCG TCG GCA GCG TCA GAT GTG TAT AAG AGA CAG CCT ACG GGN GGC WGC AG) and the reverse primer (16S_805R: GTC TCG TGG GCT CGG AGA TGT GTA TAA GAG ACA GGA CTA CHV GGG TAT CTA ATC C) with a KAPA HiFi HotStart Ready Mix (Roche, Basel, CHE). The sequencing libraries were prepared according to the 16S library preparation protocol provided by Illumina (Illumina, San Diego, CA, USA). Dual index adapters for sequencing on the Illumina MiSeq platform were attached using the Nextera XT Index kit (Illumina, San Diego, CA, USA). Each sequencing library was diluted to 5 ng/µL. We mixed equal volumes of the libraries to give a final concentration of 4 nM. The DNA concentration of the mixed libraries was measured by quantitative real-time polymerase chain reaction (qPCR) with the KAPA SYBR FAST qPCR Master mix (Roche, Basel, CHE) using primer 1 (AAT GAT ACG GCG ACC ACC) and primer 2 (CAA GCA GAA GAC GGC ATA CGA). These libraries were sequenced in a 250-bp paired-end run for V12 using the MiSeq Reagent Kit v2 (500 cycles), and in a 300-bp paired-end run for V34 using the MiSeq Reagent Kit v3 (600 cycles).

### Bioinformatics analysis

The QIIME1 analysis was performed as reported in Watanabe et al. [28] and the details are described in the following strategy: the paired-end sequencing reads were clustered by 97 % nucleotide identity, and taxonomic information was assigned using the Greengenes database (v13.8) [29] using the QIIME pipeline (v1.8.0) [26]. The data processing and assignment were performed the following steps: (1) joining of paired-end reads; (2) quality filtering with an accuracy of Q30 (> 99.9 %) and a read length > 300 bp; (3) clustering of OTUs with 97 % identity using UCLUST (v1.2.22q) [30]; (4) assigning taxonomic information to each OTU using the RDP classifier [31] with the full-length 16S rRNA gene data from Greengenes (v13.8) to determine the identity and bacterial composition. The QIIME2 analysis was performed using the following strategy: construct an ASV table with paired-end sequencing reads, and then, taxonomic information was assigned using Greengenes [29] using the QIIME2 pipeline (version 2020.2) [27]. The data processing and assignment based on the QIIME2 pipeline were performed the following steps: (1) DADA2 [32] for joining paired-end reads, filtering, and denoising; (2) assigning taxonomic information to each ASV using a naive Bayes classifier in the QIIME2 classifier with the 16S rRNA gene V2 region data for V12 sequencing and V34 region data for V34 sequencing from Greengenes to determine the identity and bacterial composition. To test two-group differences in the percentage of analyzable read numbers between V12 and V34, we calculated *p*-values using the Wilcoxon signed-rank test.

### Microbiome analysis

Alpha diversity was assessed at a depth of 30,000 reads using the OTU/ASV tables derived from the qI and qII analysis, respectively. Then, the Wilcoxon signed-rank test was performed to test the two groups diversity differences. Based on the OTU/ASV tables at the genus level, Bray-Curtis dissimilarity between the V12 and V34 regions was calculated. To assess the beta diversities of the V12 and V34 data, we performed a permutational multivariate analysis of variance (PERMANOVA). The Wilcoxon signed-rank test was performed to test differences in relative composition at the phylum and genus levels between the V12 and V34 regions, we calculated *p*-values using the Wilcoxon signed-rank test.

### qPCR analysis

qPCR was performed using KAPA SYBR Fast and LightCycler480 System II (Roche Diagnostics K.K., Rotkreuz, CHE.) under the following conditions: 95 °C for 30 s (95 °C for 5 s, 60 °C for 30 s) × 45 cycles. The primers used were *Bacteroides* (forward: CAA TCG GAG TTC TTC GTG ATA TCT A; reverse: GTT GTG AAA GTT TGC GGC TCA), *Faecalibacterium* (forward: TGT AAA CTC CTG TTG TTG AGG AAG ATA A; reverse: GCG CTC CCT TTA CAC CCA), *Bifidobacterium* (forward: CGC GTC YGG TGT GAA AG; reverse: CCC CAC ATC CAG CAT CCA), *Akkermansia* (forward: CTG AAG AAC TCG GCA CCC TT; reverse: CTT CTT CAG CTT CGG CAG GA), and bacterial 16S rRNA (forward: ACT CCT ACG GGA GGC AGC AGT; reverse: TAT TAC CGC GGC TGC TGG C). To calculate the relative abundance of each bacteria, the data were normalized by subtracting the 16S rRNA cycle threshold (Ct) value for each respective sample from the Ct values for the target bacteria to calculate ΔCt values, which are expressed as 2^ [Ct (16S PCR)-Ct (target PCR)], respectively [33, 34].

## Results

### Comparison of the 16S Profiles Using V1–V2 and V3–V4 Primers

**Fig. 1:**
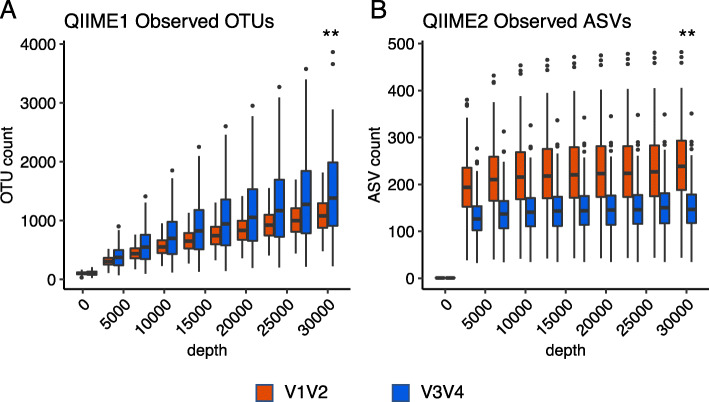
Observed operational taxonomic units (OTUs) and amplicon sequence variants (ASVs) plot at each sequencing depth for V12 (red) and V34 (blue). **A** qI and **B** qII. Statistical analysis (Wilcoxon signed-rank test) was performed at a depth of (**A**: qI) 29,998 and (**B**: qII) 30,000. Double asterisks indicate statistical significance (*p* < 0.01)

We recruited 192 volunteers, collected their stool samples, and stored them in a guanidine isothiocyanate-based reagent at ambient temperature. DNA extraction was carried out using a bead-based method as previously reported in similar studies [5–7]. The standard protocol was used for the preparation of an Illumina library to allow for the sequencing of 16S amplicons. Paired-end sequencing was performed with a 250-bp length for the V1–V2 region (V12) and with a 300-bp length for the V3–V4 region (V34). The average read counts for V12 and V34 were 44,442 and 47,220, respectively (Table 1). The average sequence qualities of V12 were above Q30 by 249 bp for read1 and by 220 bp for read2. Those of V34 were above Q30 by 244 bp for read1 and by 204 bp for read2 (Fig. 1S-A). We analyzed these raw sequence data with the standard qI and qII pipelines. The preprocessing for qI (merge, adapter trim, and primer check step) yielded analyzable read counts for V12 and V34 of 40,941 (91.3 %; V12qI) and 37,611 (79.6 %; V34qI), respectively (Table 1; Fig. 1S). In contrast, the preprocessing for qII (filtering, denoising, merging, non-chimeric step) yielded analyzable read counts of 35,609 (80.1 %; V12qII) and 31,014 (66.0 %; V34qII), respectively. Although the analyzable reads for V34 were fewer than those for V12 (*p* = 7.46e-62 (qI). *p* = 1.27e-60 (qII). Fig 1S-B,C), both datasets satisfied the typical read amount (1,000–50,000 reads) after merging and quality filtering (Table 1) [35]. The quality of the merged reads did not differ greatly between V12 and V34 (Fig. 1S-D). Based on these results, we concluded that the sequencing data obtained were of sufficient quality and quantity for microbiome analysis using both versions of the QIIME pipeline.

**Table 1:** 16S sequencing profile comparison between V12 and V34

Regions	V12	V34
	250 bp, PE	300 bp, PE
Participants	192	192
Total reads No.	8,532,874	9,066,173
Reads / Sample	44,442	47,220
Merged reads / sample	qI: 44,145 (99.3 %)	qI: 46,730 (99.0 %)
	qII: 36,639 (82.4 %)	qII: 32,843 (69.6 %)
Analyzable reads / sample	qI: 40,508 (91.0 %)	qI: 37,611 (79.6 %)
	qII: 35,609 (80.0 %)	qII: 31,014 (66.0 %)

### Difference in observed OTU/ASV

**Fig. 2:**
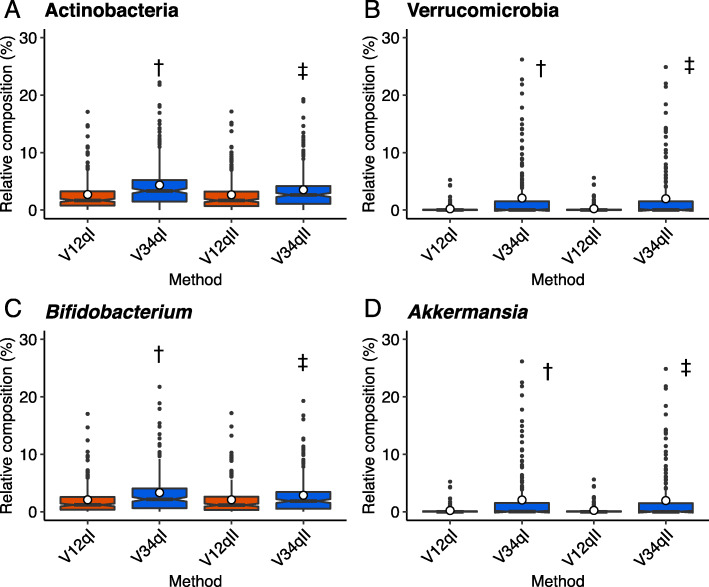
Differences in microbial structure assessed using V12 and V34. **A–D** The relative composition of the indicated phyla and genera. Daggers indicate statistical significance between V12qI and V34qI (*p* < 0.01). Double daggers indicate statistical significance between V12qII and V34qII (*p* < 0.01)

**Fig. 3:**
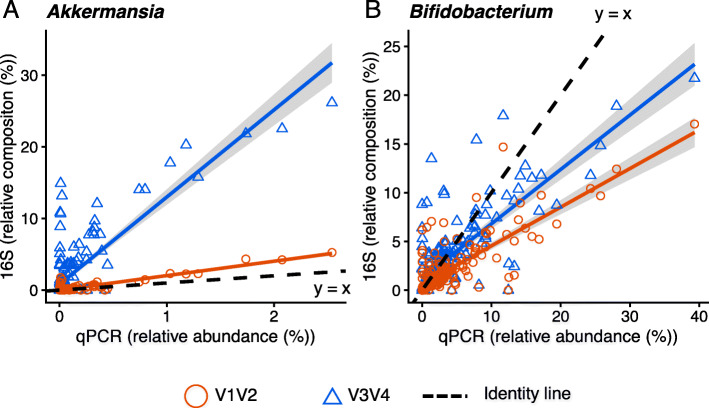
Scatter plot of the indicated genera in a comparison of the 16S analysis by qI and qPCR. The identity line (y = x) is indicated. The gray area indicates the 95 % confidence interval for each regression line

We next evaluated the alpha diversities of the V12 and V34 data. As shown in Fig. 1, at 30,000 reads, the median OTU count for V12qI (1,078) was lower than that for V34qI (1,382.8). In contrast, the median value of ASV count for V12qII (238.65) was higher than that of V34qII (147). To understand the reason for this difference, we investigated each OTU/ASV assigned by qI and qII (Fig. 2S and Table 2S). Assigned OTU/ASV rates at the phylum level were 99.46 % (V12qI), 97.76 % (V34qI), 99.84 % (V12qII), and 98.67 % (V34qII). At the genus level, these rates decreased to 69.92 % (V12qI), 64.49 % (V34qI), 63.04 % (V12qII), and 66.51 % (V34qII). The major phyla assigned were Firmicutes, Bacteroidetes, Proteobacteria, and Actinobacteria, as is commonly observed in normal gut flora. We compared both individual and total OTU counts for these major phyla and found that in the output from qI, the OTU counts of Bacteroidetes, Proteobacteria, and Actinobacteria for V34 were higher than those for V12 (Fig. 3S-A). Conversely, in the output from qII, the ASV counts of all major phyla for V34 were lower than those for V12 (Fig. 3S-B). Other than these major phyla, there were unclassified OTUs/ASVs where the highest taxonomic level assigned was only to the kingdoms Archaea or Bacteria. The use of qII reduced the counts of these unclassified ASVs to nearly zero compared to the unclassified OTUs of qI (Fig. 3S). Whether classified or unclassified, a lot of the representative sequences of OTUs are filtered out by DADA2. We labeled those sequences as filtered-out sequences. The percentage of filtered-out sequences was higher in V34 (94.3 %) than V12 (87.9 %) (Table 3S). These results indicate that both the V12 and V34 data were greatly affected by the version of QIIME used for analysis and that the combination of the V34 primer set and qII processing resulted in the detection of fewer ASVs compared to that with V12qII.

### Difference in microbial composition

To assess the beta diversities of the V12 and V34 data, we performed a nonparametric test (PERMANOVA). A Bray-Curtis dissimilarity was calculated, with the result indicating that the differences between the V12 and V34 communities were small but statistically significant (*p* = 0.001; Table 2). To further understand the specific differences between the two communities, we compared the individual bacterial compositions of V12 and V34 at the phylum level (Fig. 4S). The relative compositions of three major phyla, Bacteroidetes, Firmicutes and Proteobacteria, did not differ between V12 and V34 (Fig. 5S), while the relative compositions of Actinobacteria and Verrucomicrobia were higher for V34 than for V12 in some individuals (Fig. 2-A, -B and Fig. 6S). The mean compositions of Actinobacteria were 2.70 % (V12qI), 4.35 % (V34qI), 2.65 % (V12qII), and 3.53 % (V34qII) (Fig. 2A; Table 4S). Those of Verrucomicrobia were 0.22 % (V12qI), 2.07 % (V34qI), 0.23 % (V12qII), and 1.97 % (V34qII) (Fig. 2B; Table 4S). These differences were statistically significant between the V12 and V34 communities (Fig. 2-A, -B, and Table 4S). We further compared the relative compositions at the genus level under these two phyla. In the phylum Actinobacteria, we found a difference in the genus *Bifidobacterium*, with the relative compositions of 2.07 % (V12qI), 3.31 % (V34qI), 2.07 % (V12qII), and 2.86 % (V34qII) (Figs. 2-C and 7-S, and Table 5S-A, -B). In the phylum Verrucomicrobia, we found a difference in the genus *Akkermansia*, with the compositions of 0.22 % (V12qI), 2.05 % (V34qI), 0.23 % (V12qII), and 1.95 % (V34qII) (Figs. 2-D and 7-S, and Table5S-C, -D). These data suggest that the 16S analysis using the V34 primer set tended to estimate the higher compositions of the genera *Bifidobacterium* and *Akkermansia*.

**Table 2:** PERMANOVA results of the beta diversity calculated using Bray-Curtis dissimilarity

A	**QIIME1**	**Df**	**SumOfSqs**	**R2**	**F**	**Pr(>F)**
	V12 - V34	1	2.063104472	0.039957174	272.7097774	0.001
	Participants	191	48.12483416	0.932057689	33.3054547	0.001
	Residual	191	1.444953525	0.027985136		
	Total	383	51.63289216	1		
B	**QIIME2**	**Df**	**SumOfSqs**	**R2**	**F**	**Pr(>F)**
	V12 – V34	1	1.34266081	0.02577232	175.865223	0.001
	Participant	191	49.2961408	0.94623742	33.8059551	0.001
	Residuals	191	1.45820879	0.02799026		
	Total	383	52.0970104	1

### Comparison of the 16S analysis with quantitative analysis by qPCR

To determine whether the relative composition using V12 or V34 for the 16S analysis represents the actual relative abundance, we performed qPCR assays. We measured the bacterial DNA levels of *Akkermansia*, *Bifidobacterium*, *Bacteroides*, and *Faecalibacterium*. To examine the comparability of the qPCR and 16S analyses, we confirmed that the qPCR analysis reflected the relative composition of a mock community containing 10 bacterial species (Fig. 8S). In the 16S analysis, the relative compositions of *Bacteroides* and *Faecalibacterium* did not differ largely between the V12 and V34 data (Table 6S). We used *Bacteroides* as the major population control and *Faecalibacterium* as the minor population control. Figure 3 shows a scatter plot of the relative abundances measured by the qPCR and 16S analyses. If these values were identical, the scatter plots should be close to the identity line (y = x). Indeed, the qPCR assay for the control bacteria, *Bacteroidetes*, and *Faecalibacterium*, showed identical trends for V12 and V34 (Fig. 9S). Additionally, the slopes of the regression lines for both bacteria were nearly the same as the slope of the identity line, only with slightly higher values. In contrast, for *Akkermansia*, the regression line for V34 was far removed from the identity line even though that of V12 was nearly identical to it (Figs. 3-A and 10S-A). For *Bifidobacterium*, the regression line for V34 was closer to the identity line, but the measured values for both V12 and V34 were still different from the qPCR results (Figs. 3-B and 10S-B). These data suggest that the 16S analysis slightly underestimates the abundance of *Bifidobacterium* and overestimates the abundance of *Akkermansia* using the V34 primer set.

## Discussion

We compared the gut microbiota of 192 Japanese volunteers using both the V12 (27Fmod) and V34 regions of 16S rRNA. The amplicon sequencing method targeting the 16S rRNA gene is widely used to assess the gut bacterial composition. Therefore, it is important to be able to correctly assess its bacterial composition.

We sequenced both V12 and V34 amplicons with sufficient quality, and the number of raw reads did not differ between regions. However, after qI and qII preprocessing, the rate of decrease in the number of reads was higher in the V34 region than in the V12 region. In the OTU/ASV clustering, the number of OTUs of three major phyla, Bacteroidetes, Proteobacteria and Actinobacteria, were higher in V34 than in V12 in the qI analysis (Fig. 3S-A). In the qII analysis, the ASVs of all four major phyla, Firmicutes, Bacteriodetes, Proteobacteria and Actinobacteria, were lower in V34 than in V12 (Fig. 3S-B). Unclassified OTUs/ASVs were significantly higher in V34 than in V12 in both qI and qII. When analyzed in qII using DADA2, the number of unclassified ASVs was reduced compared to the number of unclassified OTUs with qI. DADA2 is used for ASV clustering and does not output unassigned ASVs, in contrast to OTU clustering with qI, and is reported to be superior in sequence clustering ability [32, 36, 37]. Our results indicated that the V34 results contain more filtered-out sequences that would be excluded when processed by DADA2, which clusters ASVs more accurately.

Chen et al. claimed that the V34 region was better suited than the V12 region for the analysis of the human gut microbiota because V12 “failed to detect” the majority of Bifidobacteriales [20]. Similarly, Graspeuntner et al. indicated that some specific bacteria, especially the phylum of Actinobacteria, “were not represented in V12” in the analysis of the female genital tract microbiome [24]. However, they did not use the primer (27Fmod) developed by Kim et al. [21] that dramatically improves the detection of *Bifidobacterium* by approximately 41-fold compared to that with 27F, and thus, it was necessary to perform another comparison of these regions using the improved primer set. Chen et al. reported an average composition of 0.03 % for Bifidobacteriales. In the present study, the relative composition of the genus *Bifidobacterium* based on the V12 with 27Fmod was on average 2.1 %, although it was lower than that based on the V34 region (3.3 %). We again confirmed that the use of V12 with 27Fmod improved the detection of *Bifidobacterium* dramatically (2.1 %, 70-fold) over the standard V12 (0.03 %). However, because both V12 and V34, for the 16S analysis, were estimated to have lower composition compared to those with qPCR targeting the 16S rRNA gene, caution might still be needed when evaluating the phylum of Actinobacteria, and especially the genus *Bifidobacterium*, using any region.

*Akkermansia*, belonging to the phylum Verrucomicrobia, is considered an important health indicator due to its associations with obesity and diabetes [38, 39]. This study demonstrated that *Akkermansia* was detected at a relative abundance close to that with qPCR based on V12 but was largely dissociated with V34. Sometimes, a high copy number of rRNA in the genome causes such discrepancies between the 16S analysis and others [40–42]. In the qPCR analysis, we used a primer for the *rplL* gene of *Akkermansia*, which is known as a single copy housekeeping gene. Recently the genome of *Akkermansia* originating from a Japanese individual has been reported, indicating that this strain has three copies of rRNA, which is in the normal range of rRNA copy numbers (rrnDB v.5.6 [41], date accessed 19 Jan 2021). Another possibility is that some bacteria have a high similarity only in the V34 region, but not in the V12 one. One example of such bacteria is *Cronobacter* whose V34 sequence shows 99.32 % similarity with that of *Akkermansia* while the corresponding V12 sequence only shows 71.43 % similarity (Fig. 11S). Indeed, 32 % of the OTUs assigned as *Akkermansia* by V34 were matched to *Cronobacter* through BLAST similarity search. However, the sequence used for *Cronobacter* is from draft genome data. Also, only one genome for *Akkermansia* is available from the Japanese population. The lack of these genomic data is an obstacle in determining the cause of the large difference between qPCR/V12 and V34. Future genomic studies of minor gut bacteria will be needed to uncover the cause of this discrepancy.

This study revealed that *Akkermansia* could be detected in 45 % (V12qI), 34 % (V12qII), 66 % (V34qI), and 40 % (V34qII) of 192 participants. Their relative composition (interquartile range) was 0.00–0.08 % (V12qI), 0.00–1.51 % (V34qI), 0.00–0.09 % (V12qII), and 0.00–1.50 % (V34qII). Previous studies have shown that Asians (V12 and V13) have a lower composition of Verrucomicrobia than Americans (V13 and V2) and Europeans (full-length 16S rRNA gene; Columbia: 1.2 % ± 4.2, USA: 0.1 % ± 0.2, Europe: 1.2 % ± 2.6, Japan: 0.0 % ± 0.1, South Korea: 0.0 % ± 0.0) [43] and that the relative composition of *Akkermansia* is 0.05–3.24 % (V34) in Japanese individuals [44]. Our data were similar to these results. Because *Akkermansia* seems to be present in the human gut with the low composition, we believe that the difference in the relative composition between V12 and V34 could not have been observed unless a large enough sample size was analyzed.

The composition of the human gut microbiota has been reported to vary by nationality and ethnicity [45, 46]. For example, the Japanese gut microbiota is characterized by a higher composition of *Bifidobacterium* [45, 46]. We used only feces from Japanese donors in this study, which makes our analysis less racially diverse. Therefore, the results of this study alone might not provide a complete picture of the effect of regional selection, especially for bacteria that are found only in other populations or in the feces of non-human animals. Additional comparisons based on mock communities with known composition or with feces of various origins might be informative.

## Conclusions

The data derived from the V34 region showed that the bacterial composition differed from the actual abundance, especially for the genus *Akkermansia*, and that it contains more unclassified and filtered-out sequences. These results indicate that the bacterial composition derived from the V34 region might differ from the actual abundance for specific gut bacteria and suggest that the V12 (27Fmod) region is more suitable for analyzing the Japanese intestinal microbiome.

## Supplementary Information

**Additional file 1: Figure 1S.** Comparison of (A) quality plots of raw reads, (B,C) percentage of analyzable reads between (B) V12qI and V34qI and (C) V12qII and V34qII, and (D) quality plots and length distribution of analyzable reads. (A,D) The mean quality scores are plotted. The y-axis on the graph shows the quality scores. The higher the score, the better the base call. The background of the graph divides the Q-score into good: Q > 28 (green), passable: 28 > Q > 20 (orange) and poor: 20 > Q (red). (B,C) The ratio of the number of analyzable reads to the number of raw reads is shown. Double asterisks indicate statistical significance (*p *< 0.01). **Figure 2S.** Percentage of classified operational taxonomic units (OTUs) and amplicon sequence variants (ASVs). At each classification level, the results of calculating the percentage of OTUs/ASVs assigned to a taxonomy in relation to the total number of OTUs/ASVs are shown. **Figure 3S.** (A,B) Differences in the operational taxonomic unit (OTU) and amplicon sequence variant (ASVs) counts for the indicated phylum between the V12 and V34 regions. Double asterisks indicate statistical significance (*p *< 0.01). (C,D) Comparison of the total OTU/ASV numbers for the indicated phylum. Unclassified OTUs/ASVs are included in k__Bacteria;__, k__Bacteria;p__, k__Archaea;__, and Unclassified;__. **Figure 4S.** Bar chart of the individual bacterial compositions using V12 and V34 at the phylum level using (A) qI (upper panel: V12; lower panel: V34) and (B) qII (upper panel: V12; lower panel: V34). **Figure 5S.** Relative composition of Bacteroidetes, Firmicutes, and Proteobacteria using V12 and V34. **Figure 6S.** Bar chart of the individual bacterial compositions using V12 and V34 for the indicated phyla using (A) qI (upper panel: V12. lower panel V34) and (B) qII (upper panel: V12; lower panel: V34). **Figure 7S.** Bar chart of the individual bacterial compositions using V12 and V34 for the indicated genera using (A) qI (upper panel: V12; lower panel: V34) and (B) qII (upper panel: V12; lower panel: V34). **Figure 8S.** Bar chart of bacterial relative abundance using a DNA mock community kindly provided by NITE (National Institute of Technology and Evaluation, Tokyo, JPN). This community is made from an equal mix of genomic data from the 10 indicated strains. qPCR was performed targeting the *rplL *gene of each bacteria and normalized by total bacteria using the measured value of 16S rRNA gene. rRNA copy num indicates the percentage of copy number of the 16S rRNA gene. V12 and V34 indicate the results of 16S analysis. **Figure 9S.** Scatter plot of the indicated genera to compare the 16S analysis by (A,B) qI, (C,D) qII, and qPCR. The identity line (y = x) is indicated. The gray area indicates the 95% confidence interval for each regression line. **Figure 10S.** Scatter plot of the indicated genera to compare the 16S analysis by qII and qPCR. The identity line (y = x) is indicated. The gray area indicates the 95% confidence interval for each regression line. **Figure 11S.** Alignments and similarity of *Cronobacter *and OTU representative sequence with *Akkermansia *for the 16S rRNA gene. (A) V12 (B) V34 of 16S rRNA gene. *Akkermansia *indicates reference sequences, which are derived from *Akkermansia muciniphila *strain JCM 30893. *Cronobacter *sequences are derived from *Cronobacter sakazakii *strain cro360A2. Observed_OTU sequence is a representative sequence assigned to *Akkermansia*, which was matched to *Cronobacter*through BLAST. The values of similarity indicate percent identity between the reference sequence and the query sequence calculated by BLAST.

**Additional file 2:** **Table 1S.** Distribution of the selected participants. **Table 2S.** Percentage of classified OTUs/ASVs at each classification level. **Table 3S.** OTUs/ASVs numbers and the percentage of filtered-out sequences. **Table 4S.** Lists of bacteria that showed statistical differences at the phylum level between V12 and V34. **Table 5S.** Lists of bacteria that showed statistical differences at the genus level between V12 and V34. **Table 6S.** Average compositions of the genera *Bacteroides *and *Faecalibacterium*.

**Additional file 3.** Relative abundance of *Akkermansia*, *Bifidobacterium*, *Bacteroides*, and *Faecalibacterium* by qPCR. To calculate the relative abundance of each bacteria, the data were normalized by subtracting the 16S rRNA cycle threshold (Ct) value for each respective sample from the Ct values for the target bacteria to calculate ΔCt values, which are expressed as 2^ [Ct (16S PCR)-Ct (target PCR)], respectively.

## Data Availability

The sequencing datasets generated during the current study are available in the NCBI repository, accession numbers SRR13985619-SRR13986002 in PRJNA715083 (https://www.ncbi.nlm.nih.gov/bioproject/PRJNA715083). The data of each bacterial relative abundance obtained by qPCR is attached as an Additional file 3.

## Abbreviations

16S: 16S rRNA gene
V12: V1–V2 region
V34: V3–V4 region
OTU: Operational taxonomic unit
ASV: Amplicon sequence variant
qI: QIIME1
qII: QIIME2
PCoA: Principal coordinates analysis
PERMANOVA: Permutational multivariate analysis of variance
qPCR: Quantitative real-time polymerase chain reaction
