# Supplementary material for: Benchmark of 16S rRNA gene amplicon sequencing using Japanese gut microbiome data from the V1–V2 and V3–V4 primer sets
Source: BMC Genomics. 2021 Jul 10;22:527. doi: 10.1186/s12864-021-07746-4 (PMC8272389; doi:10.1186/s12864-021-07746-4)
Supplement: Supplementary file 1 — Additional file 1: Figure 1S. Comparison of (A) quality plots of raw reads, (B,C) percentage of analyzable reads between (B) V12qI and V34qI and (C) V12qII and V34qII, and (D) quality plots and length distribution of analyzable reads. (A,D) The mean quality scores are plotted. The y-axis on the graph shows the quality scores. The higher the score, the better the base call. The background of the graph divides the Q-score into good: Q > 28 (green), passable: 28 > Q > 20 (orange) and poor: 20 > Q (red). (B,C) The ratio of the number of analyzable reads to the number of raw reads is shown. Double asterisks indicate statistical significance (p < 0.01). Figure 2S. Percentage of classified operational taxonomic units (OTUs) and amplicon sequence variants (ASVs). At each classification level, the results of calculating the percentage of OTUs/ASVs assigned to a taxonomy in relation to the total number of OTUs/ASVs are shown. Figure 3S. (A,B) Differences in the operational taxonomic unit (OTU) and amplicon sequence variant (ASVs) counts for the indicated phylum between the V12 and V34 regions. Double asterisks indicate statistical significance (p < 0.01). (C,D) Comparison of the total OTU/ASV numbers for the indicated phylum. Unclassified OTUs/ASVs are included in k__Bacteria;__, k__Bacteria;p__, k__Archaea;__, and Unclassified;__. Figure 4S. Bar chart of the individual bacterial compositions using V12 and V34 at the phylum level using (A) qI (upper panel: V12; lower panel: V34) and (B) qII (upper panel: V12; lower panel: V34). Figure 5S. Relative composition of Bacteroidetes, Firmicutes, and Proteobacteria using V12 and V34. Figure 6S. Bar chart of the individual bacterial compositions using V12 and V34 for the indicated phyla using (A) qI (upper panel: V12. lower panel V34) and (B) qII (upper panel: V12; lower panel: V34). Figure 7S. Bar chart of the individual bacterial compositions using V12 and V34 for the indicated genera using (A) qI (upper panel: V12; lower panel: V34 [file 12864_2021_7746_MOESM1_ESM.pdf]

Benchmark of 16S rRNA gene amplicon sequencing using Japanese gut microbiome data from the V1–V2 and V3–V4 primer sets

Shoichiro Kameoka[a,e], Daisuke Motooka[a,b,d], Satoshi Watanabe[e], Ryuichi Kubo[e], Nicolas Jung[a], Yuki Midorikawa[e], Natsuko O. Shinozaki[e], Yu Sawai[e], Aya K. Takeda[e], and Shota Nakamura[a,b,c,d]#

[a]Department of Infection Metagenomics, Genome Information Research Center, Research Institute for Microbial Diseases, Osaka University, Suita, Osaka, Japan

[b]Next-Generation Sequencing Core Facility, Genome Information Research Center, Research Institute for Microbial Diseases, Osaka University, Suita, Osaka, Japan

[c]Laboratory of Pathogen Detection and Identification, International Research Center for Infectious Diseases, Research Institute for Microbial Diseases, Osaka University, Suita, Osaka, Japan

[d]Integrated Frontier Research for Medical Science Division, Institute for Open and Transdisciplinary Research Initiatives, Osaka University, Suita, Osaka, Japan

[e]Cykinso, Inc. Shibuya, Tokyo, Japan

#Address correspondence to Shota Nakamura, nshota@gen-info.osaka-u.ac.jp

Supplementary Information

Fig. 1S Comparison of (A) quality plots of raw reads, (B,C) percentage of analyzable reads between (B) V12qI and V34qI and (C) V12qII and V34qII, and (D) quality plots and length distribution of analyzable reads. (A,D) The mean quality scores are plotted. The y-axis on the graph shows the quality scores. The higher the score, the better the base call. The background of the graph divides the Q-score into good: $Q > 28$ (green), passable: $28 > Q > 20$ (orange) and poor: $20 > Q$ (red). (B,C) The ratio of the number of analyzable reads to the number of raw reads is shown. Double asterisks indicate statistical significance ($p < 0.01$).

Fig. 2S Percentage of classified operational taxonomic units (OTUs) and amplicon sequence variants (ASVs). At each classification level, the results of calculating the percentage of OTUs/ASVs assigned to a taxonomy in relation to the total number of OTUs/ASVs are shown.

Fig. 3S (A,B) Differences in the operational taxonomic unit (OTU) and amplicon sequence variant (ASVs) counts for the indicated phylum between the V12 and V34 regions. Double asterisks indicate statistical significance ($p < 0.01$). (C,D) Comparison of the total OTU/ASV numbers for the indicated phylum. Unclassified OTUs/ASVs are included in k__Bacteria;__, k__Bacteria;p__, k__Archaea;__, and Unclassified;__.

Fig. 4S Bar chart of the individual bacterial compositions using V12 and V34 at the phylum level using (A) qI (upper panel: V12; lower panel: V34) and (B) qII (upper panel: V12; lower panel: V34).

Fig. 5S Relative composition of Bacteroidetes, Firmicutes, and Proteobacteria using V12 and V34.

Fig. 6S Bar chart of the individual bacterial compositions using V12 and V34 for the indicated phyla using (A) qI (upper panel: V12. lower panel V34) and (B) qII (upper panel: V12; lower panel: V34).

Fig. 7S Bar chart of the individual bacterial compositions using V12 and V34 for the indicated genera using (A) qI (upper panel: V12; lower panel: V34) and (B) qII (upper panel: V12; lower panel: V34).

Fig. 8S Bar chart of bacterial relative abundance using a DNA mock community kindly provided by NITE (National Institute of Technology and Evaluation, Tokyo, JPN). This community is made from an equal mix of genomic data from the 10 indicated strains. qPCR was performed targeting the

rplL gene of each bacteria and normalized by total bacteria using the measured value of 16S rRNA gene. rRNA copy num indicates the percentage of copy number of the 16S rRNA gene. V12 and V34 indicate the results of 16S analysis.

Fig. 9S Scatter plot of the indicated genera to compare the 16S analysis by (A,B) qI, (C,D) qII, and qPCR. The identity line (y = x) is indicated. The gray area indicates the 95% confidence interval for each regression line.

Fig. 10S Scatter plot of the indicated genera to compare the 16S analysis by qII and qPCR. The identity line (y = x) is indicated. The gray area indicates the 95% confidence interval for each regression line.

Fig. 11S Alignments and similarity of *Cronobacter* and OTU representative sequence with *Akkermansia* for the 16S rRNA gene. (A) V12 (B) V34 of 16S rRNA gene. *Akkermansia* indicates reference sequences, which are derived from *Akkermansia muciniphila* strain JCM 30893. *Cronobacter* sequences are derived from *Cronobacter sakazakii* strain cro360A2. Observed_OTU sequence is a representative sequence assigned to *Akkermansia*, which was matched to *Cronobacter*

through BLAST. The values of similarity indicate percent identity between the reference sequence and the query sequence calculated by BLAST.

Fig 1S

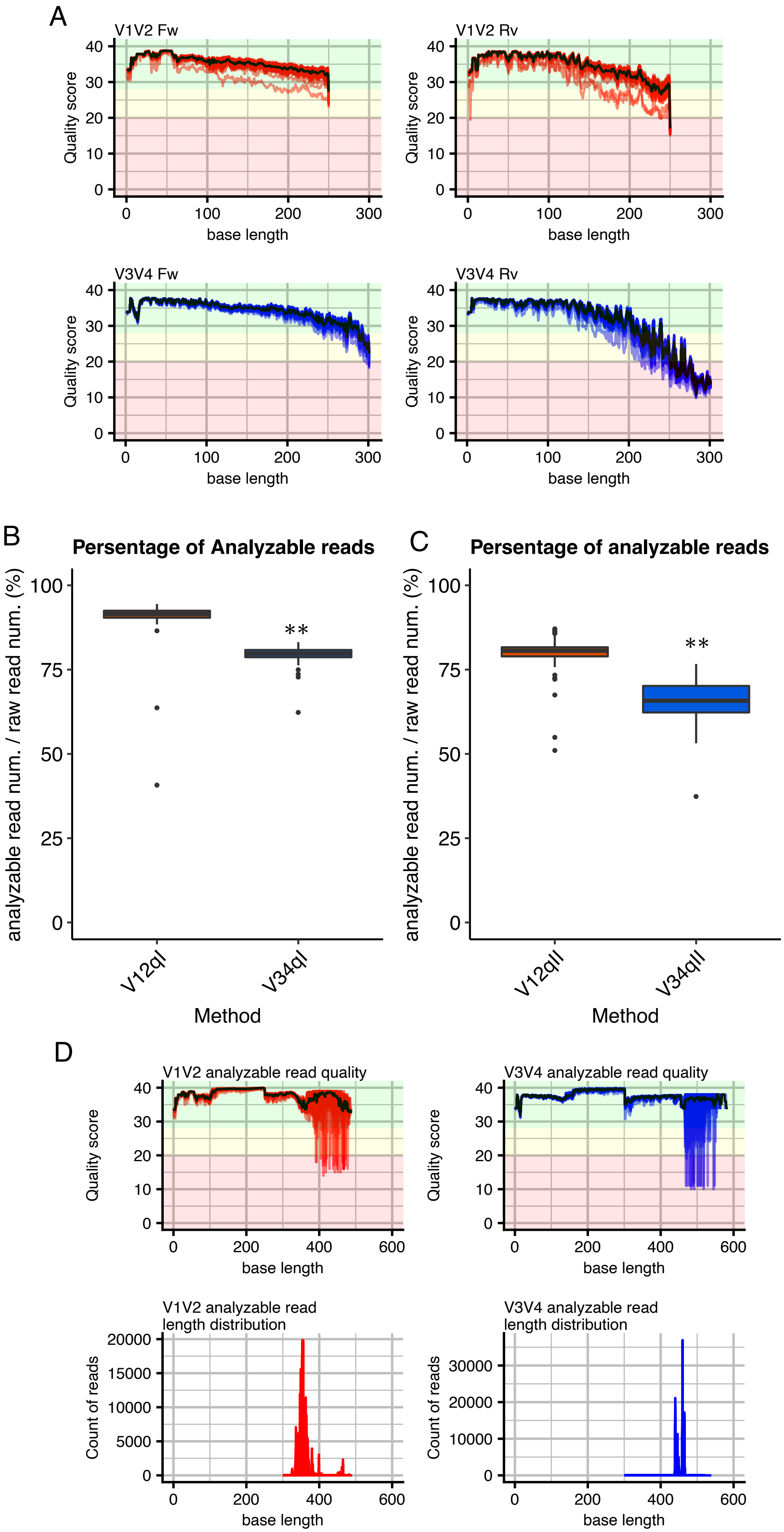

A
V1V2 Fw
V1V2 Rv
V3V4 Fw
V3V4 Rv
Quality score
base length
B
Persentage of Analyzable reads
C
Persentage of analyzable reads
analyzable read num. / raw read num. (%)
**
V12qI
V34qI
V12qII
V34qII
Method
D
V1V2 analyzable read quality
V3V4 analyzable read quality
V1V2 analyzable read length distribution
V3V4 analyzable read length distribution
Count of reads

Fig 2S

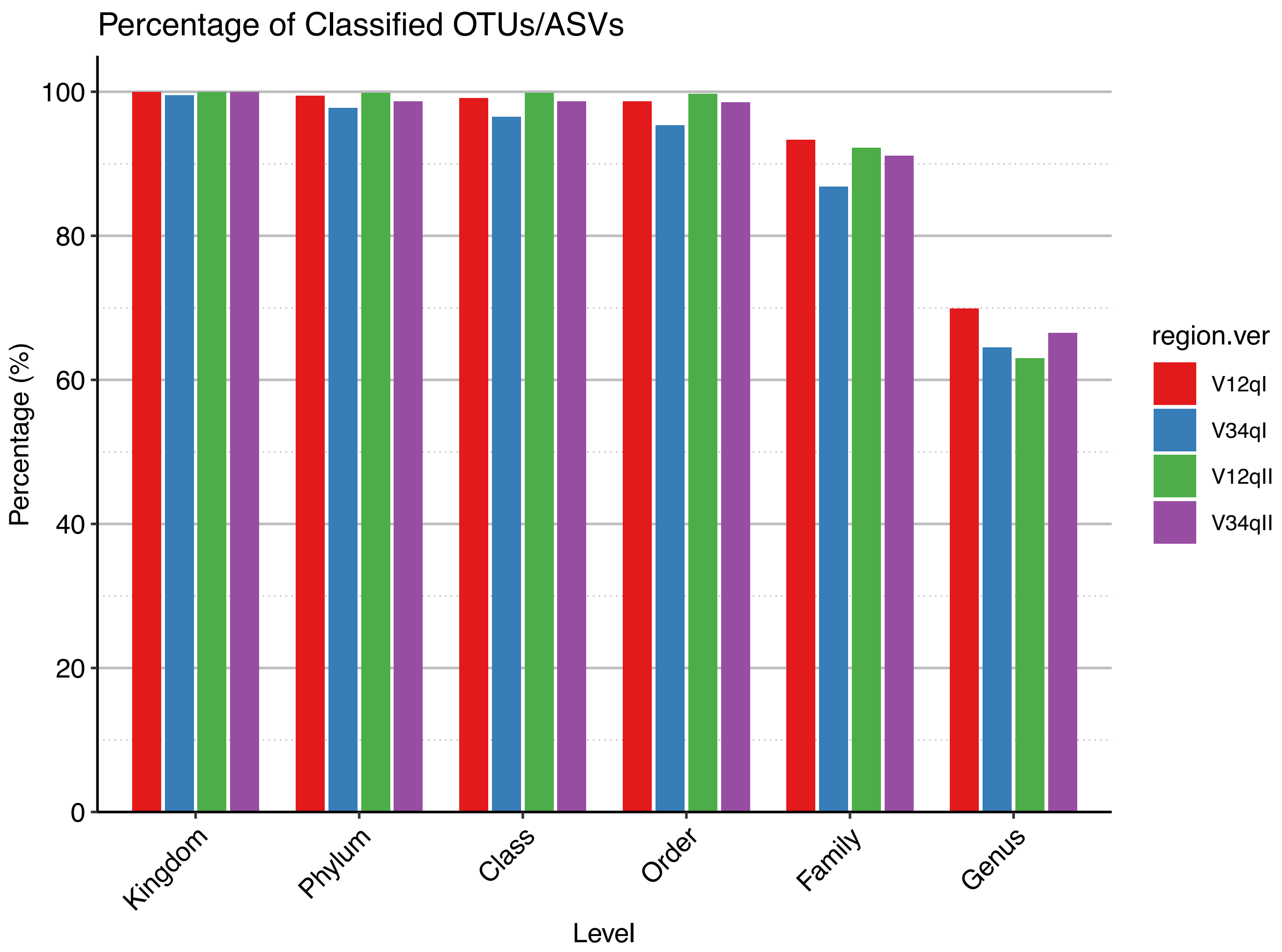
Percentage of Classified OTUs/ASVs
100
80
60
40
20
0
Percentage (%)
Kingdom
Phylum
Class
Order
Family
Genus
Level
region.ver
V12qI
V34qI
V12qII
V34qII

Fig 3S

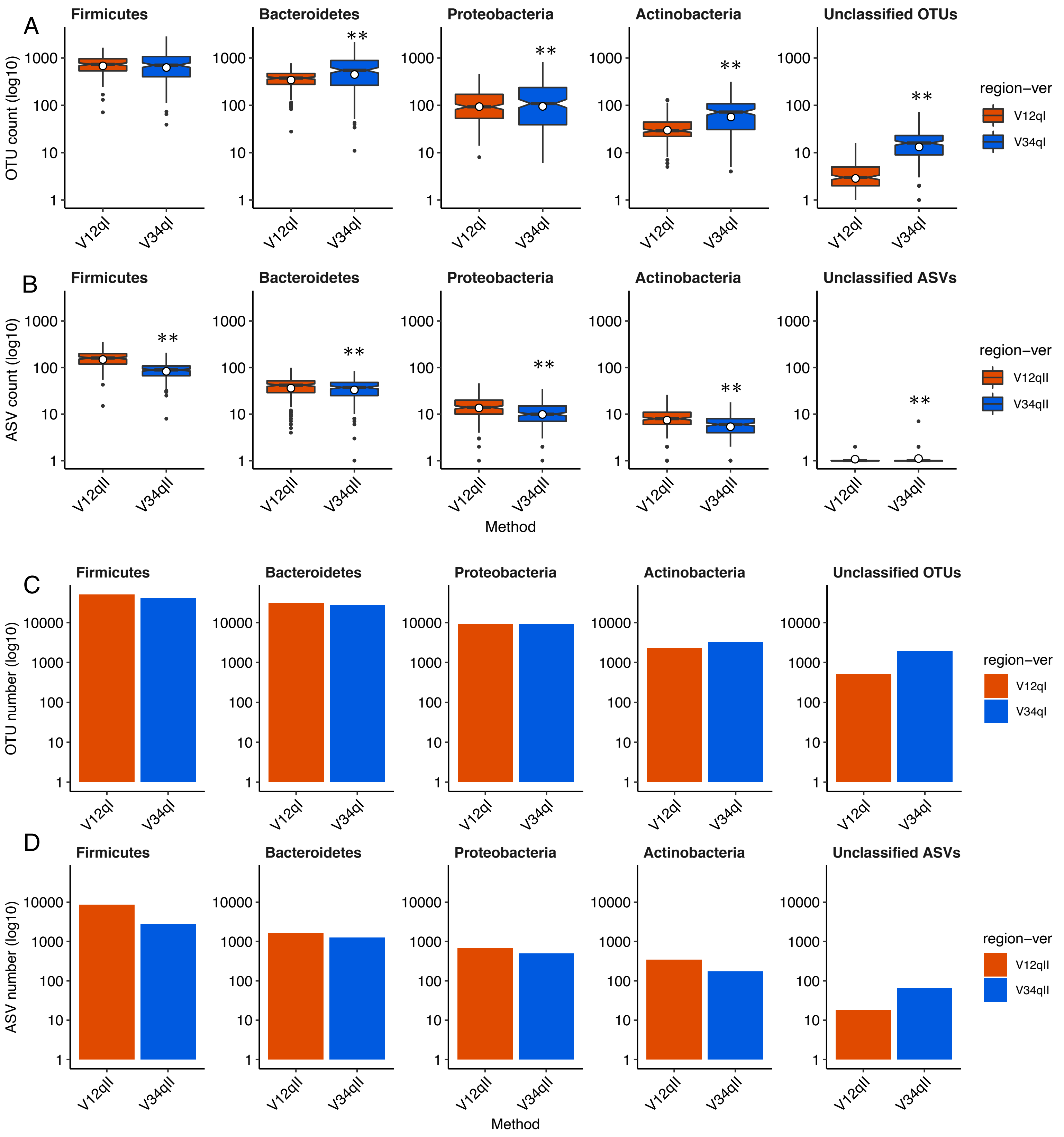
A
B
C
D
Firmicutes
Bacteroidetes
Proteobacteria
Actinobacteria
Unclassified OTUs
Unclassified ASVs
OTU count (log10)
ASV count (log10)
OTU number (log10)
ASV number (log10)
Method
region−ver
V12ql
V34ql
V12qll
V34qll
**

Fig 4S

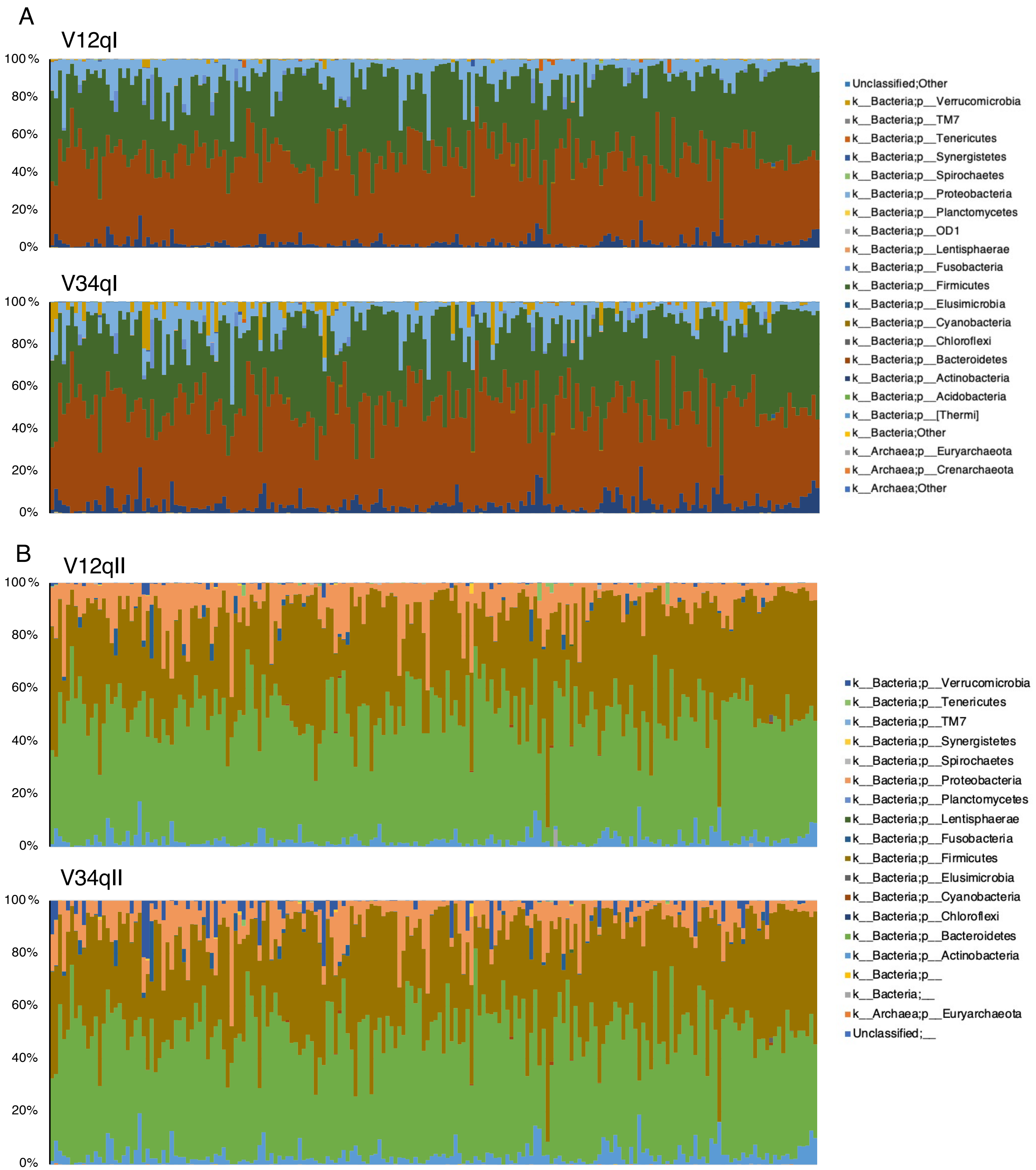
A
V12qI
100 %
80%
60%
40%
20%
0%
Unclassified;Other
k__Bacteria;p__Verrucomicrobia
k__Bacteria;p__TM7
k__Bacteria;p__Tenericutes
k__Bacteria;p__Synergistetes
k__Bacteria;p__Spirochaetes
k__Bacteria;p__Proteobacteria
k__Bacteria;p__Planctomycetes
k__Bacteria;p__OD1
k__Bacteria;p__Lentisphaerae
k__Bacteria;p__Fusobacteria
k__Bacteria;p__Firmicutes
k__Bacteria;p__Elusimicrobia
k__Bacteria;p__Cyanobacteria
k__Bacteria;p__Chloroflexi
k__Bacteria;p__Bacteroidetes
k__Bacteria;p__Actinobacteria
k__Bacteria;p__Acidobacteria
k__Bacteria;p__[Thermi]
k__Bacteria;Other
k__Archaea;p__Euryarchaeota
k__Archaea;p__Crenarchaeota
k__Archaea;Other
V34qI
B
V12qII
k__Bacteria;p__Verrucomicrobia
k__Bacteria;p__Tenericutes
k__Bacteria;p__TM7
k__Bacteria;p__Synergistetes
k__Bacteria;p__Spirochaetes
k__Bacteria;p__Proteobacteria
k__Bacteria;p__Planctomycetes
k__Bacteria;p__Lentisphaerae
k__Bacteria;p__Fusobacteria
k__Bacteria;p__Firmicutes
k__Bacteria;p__Elusimicrobia
k__Bacteria;p__Cyanobacteria
k__Bacteria;p__Chloroflexi
k__Bacteria;p__Bacteroidetes
k__Bacteria;p__Actinobacteria
k__Bacteria;p__
k__Bacteria;__
k__Archaea;p__Euryarchaeota
Unclassified;__
V34qII

Fig 5S

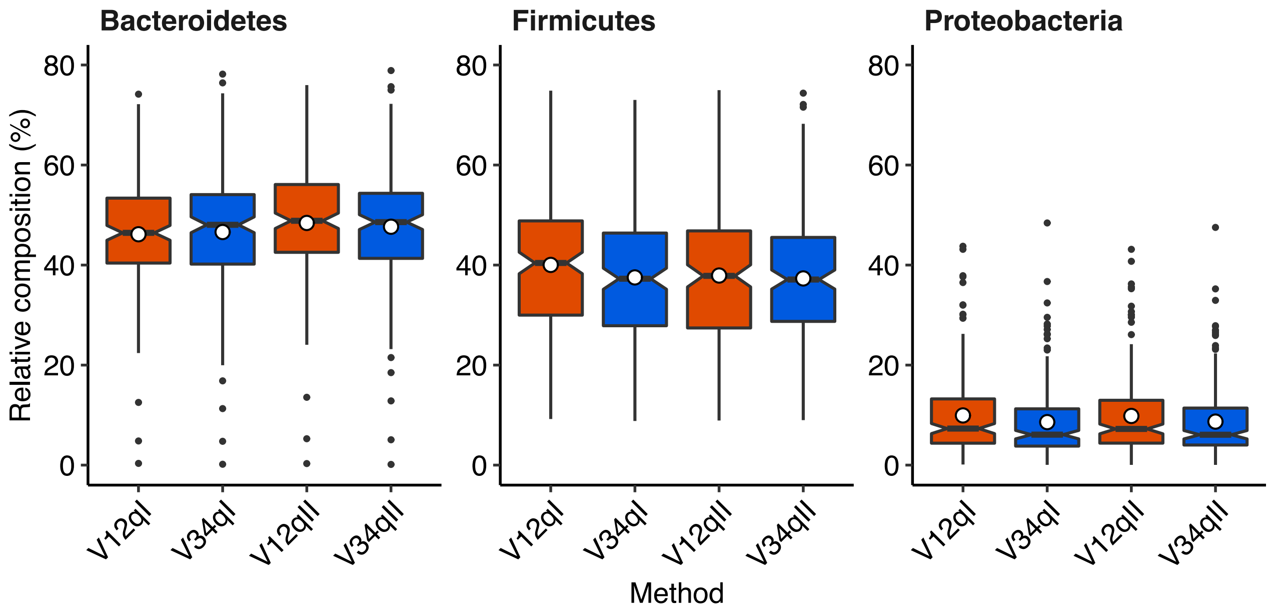
Bacteroidetes
Firmicutes
Proteobacteria
Relative composition (%)
0
20
40
60
80
V12qI
V34qI
V12qII
V34qII
Method

Fig 6S

A

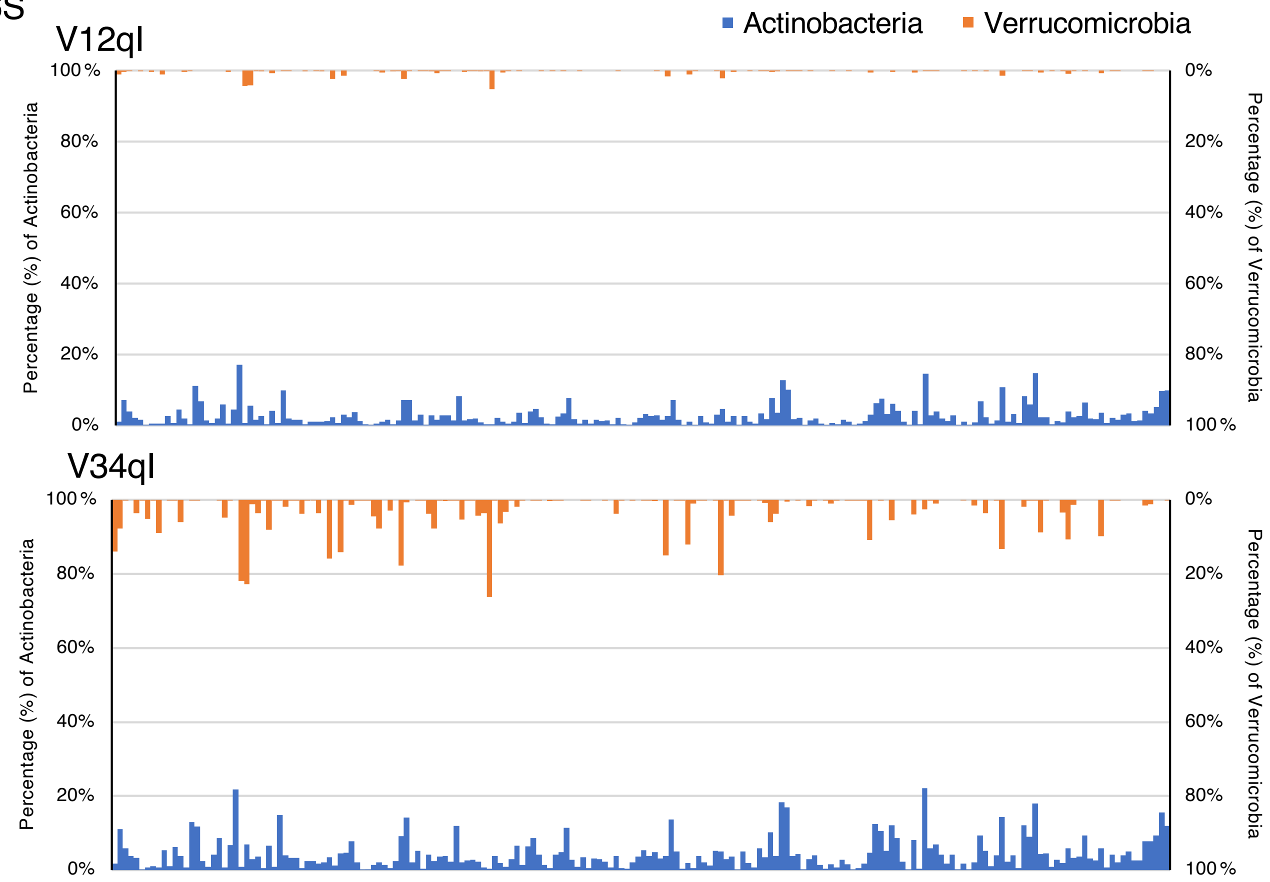
Actinobacteria
Verrucomicrobia
V12qI
V34qI
Percentage (%) of Actinobacteria
Percentage (%) of Verrucomicrobia
100 %
80%
60%
40%
20%
0%

B

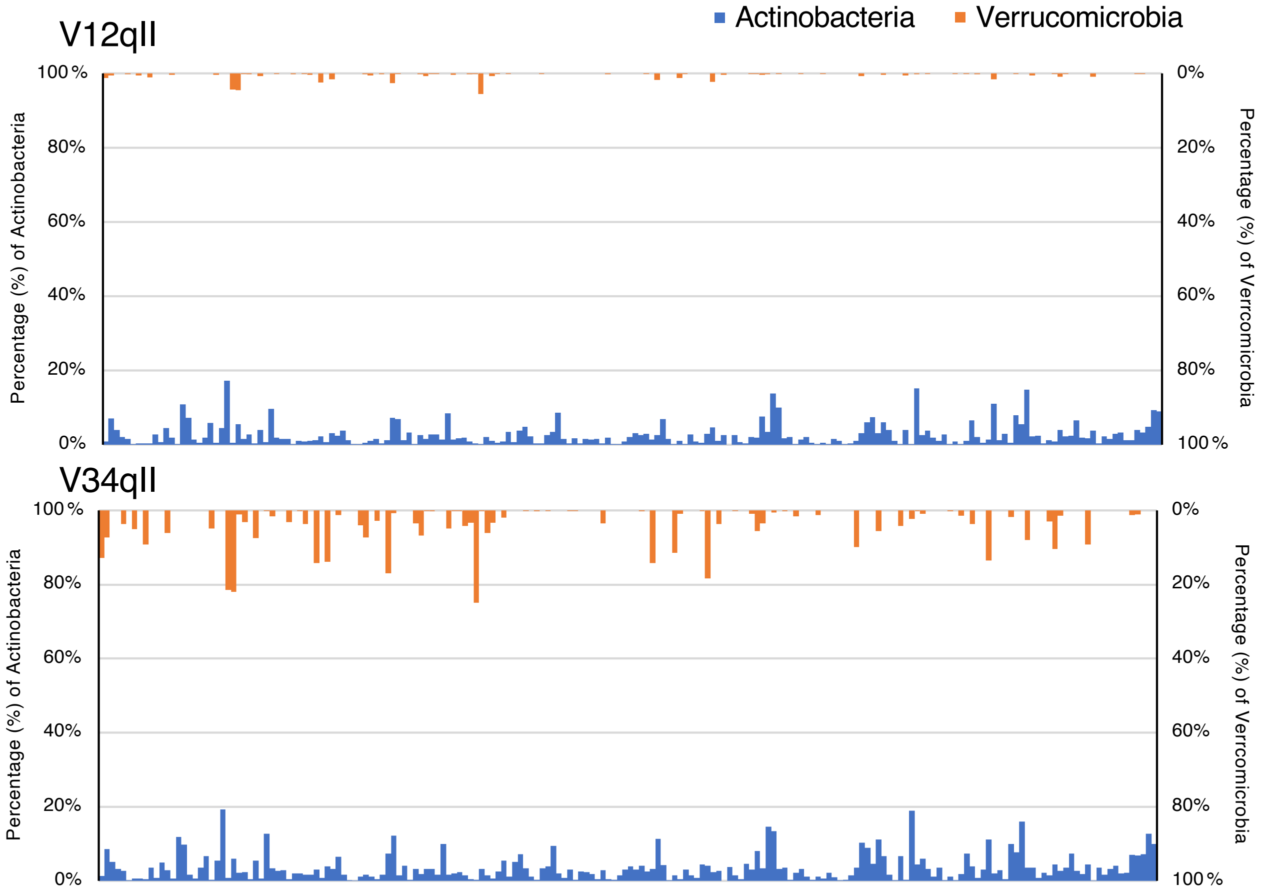
Actinobacteria
Verrucomicrobia
V12qII
V34qII
Percentage (%) of Actinobacteria
Percentage (%) of Verrcomicrobia
100 %
80%
60%
40%
20%
0%

Fig 7S

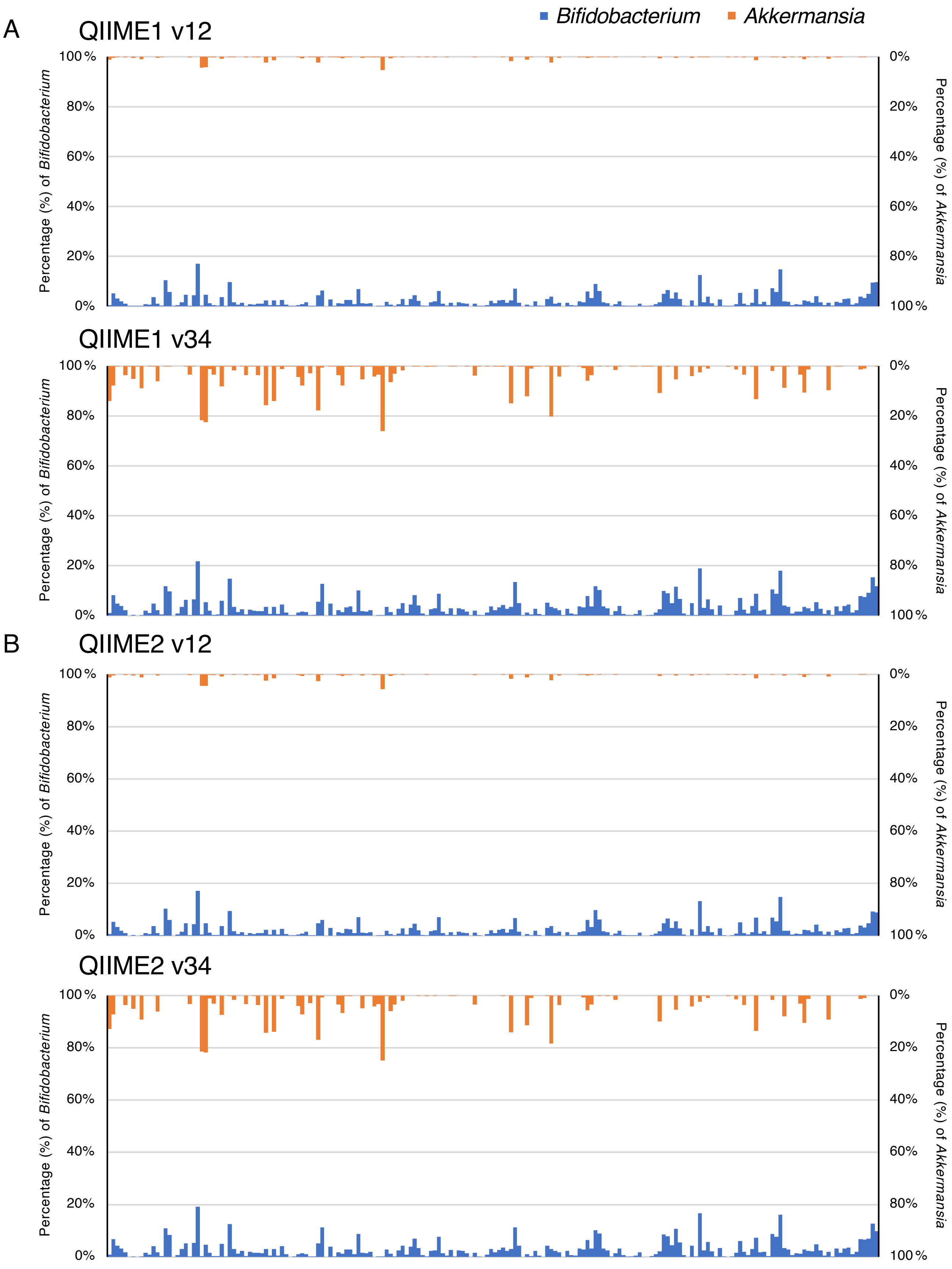

Bifidobacterium
Akkermansia
A
QIIME1 v12
QIIME1 v34
B
QIIME2 v12
QIIME2 v34
Percentage (%) of Bifidobacterium
Percentage (%) of Akkermansia
100%
80%
60%
40%
20%
0%

Fig 8S

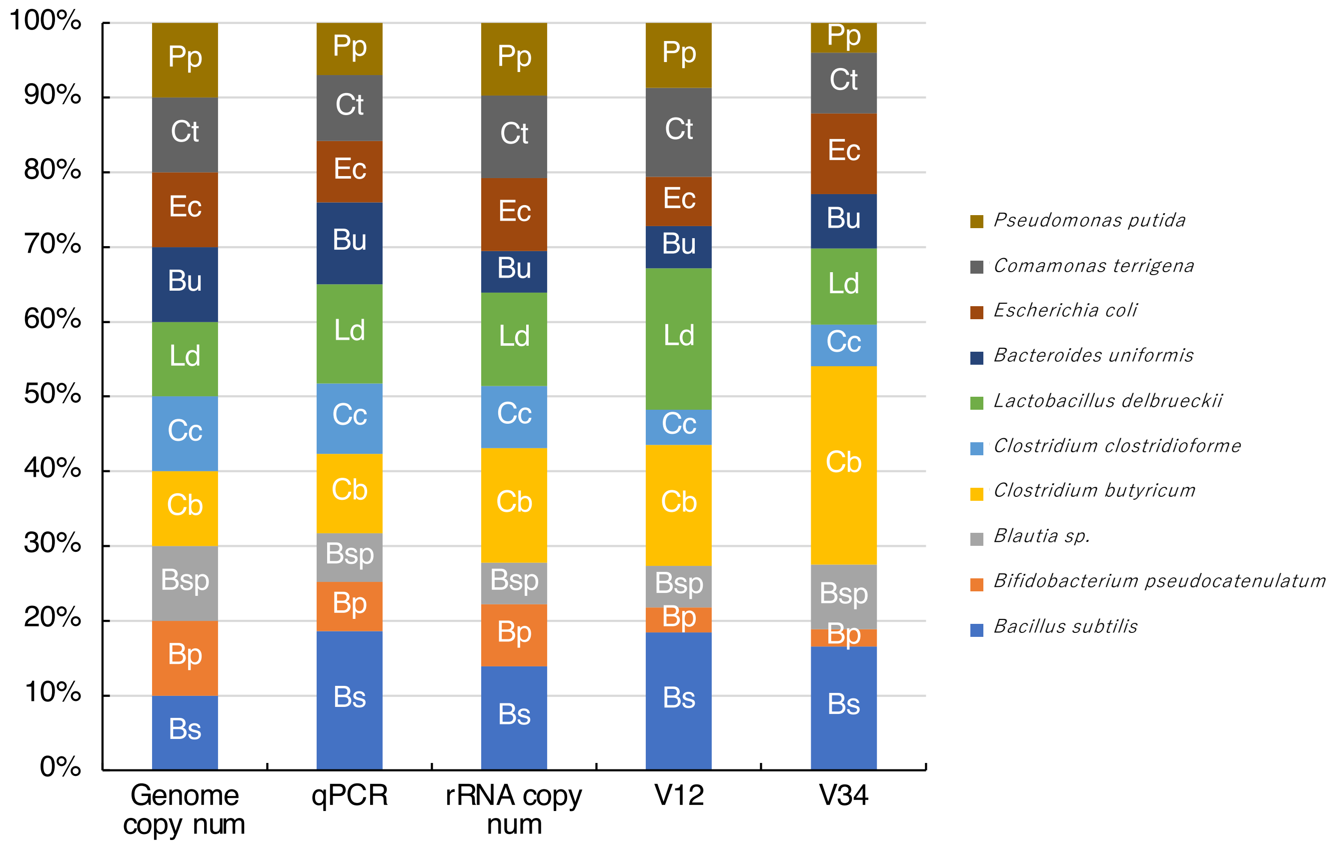
100%
90%
80%
70%
60%
50%
40%
30%
20%
10%
0%
Genome copy num
qPCR
rRNA copy num
V12
V34
Pp
Ct
Ec
Bu
Ld
Cc
Cb
Bsp
Bp
Bs
Pseudomonas putida
Comamonas terrigena
Escherichia coli
Bacteroides uniformis
Lactobacillus delbrueckii
Clostridium clostridioforme
Clostridium butyricum
Blautia sp.
Bifidobacterium pseudocatenulatum
Bacillus subtilis

Fig 9S

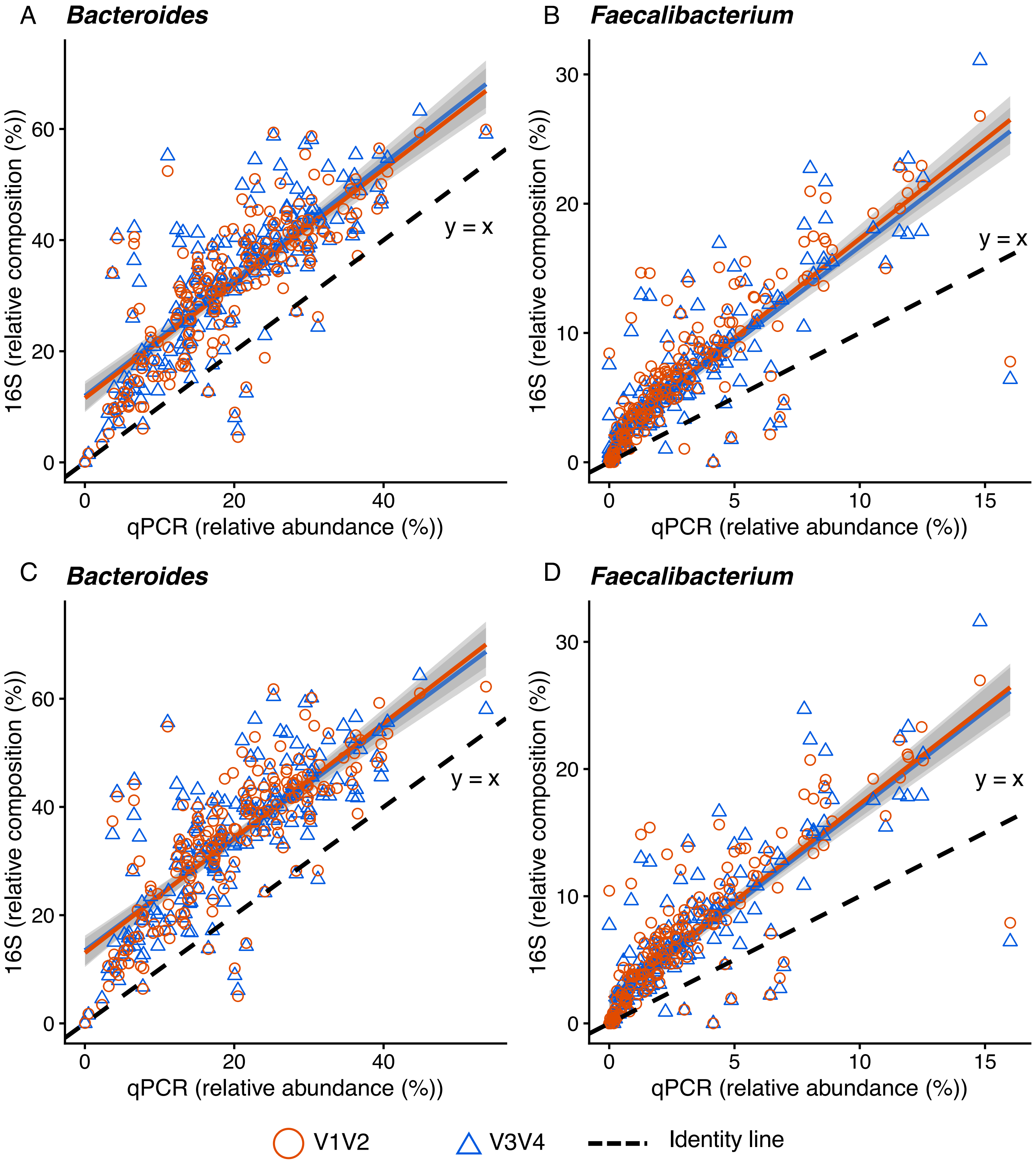
A
Bacteroides
B
Faecalibacterium
C
Bacteroides
D
Faecalibacterium
16S (relative composition (%))
qPCR (relative abundance (%))
y = x
0
20
40
60
0
5
10
15
30
V1V2
V3V4
Identity line

Fig 10S

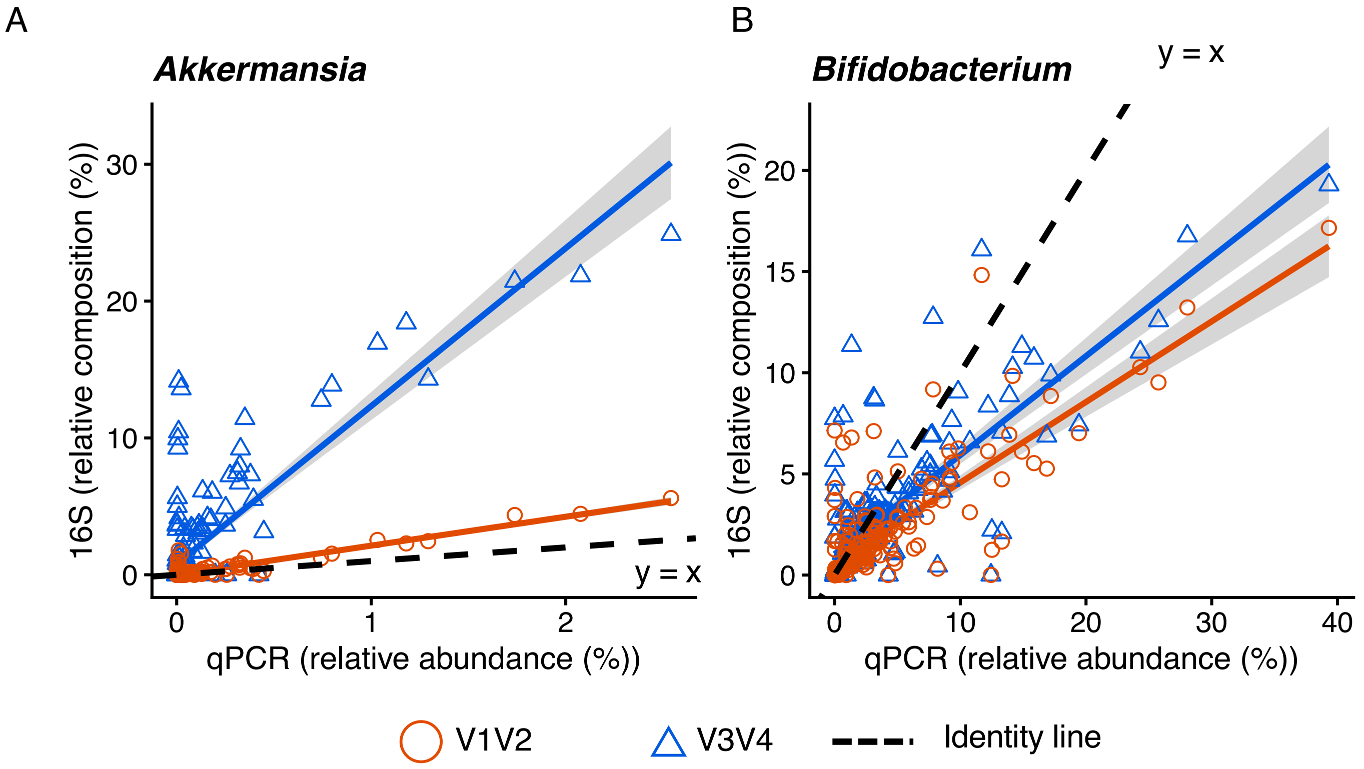
A
Akkermansia
16S (relative composition (%))
0
10
20
30
qPCR (relative abundance (%))
0
1
2
y = x
B
Bifidobacterium
16S (relative composition (%))
0
5
10
15
20
qPCR (relative abundance (%))
0
10
20
30
40
y = x
V1V2
V3V4
Identity line

Fig 11S

A

```
V12
Akkermansia    1 AGAGTTTGATTCTGGCTCAGAACGAACGCTGGCGGCGTGGATAAGACATGCAAGTCGAAC
Cronobacter    1 ..........CA.........TT.............AG.CC...C...............
consensus      1 **********..*********..*************..*..***.***************

Akkermansia   61 GA-GAGAATTGCTAGCTTGCTAAT-AATTCTCTAGTGGCGCACGGGTGAGTAACACGTGA
Cronobacter   61 .GTA.C.GGGAGC........GC.CTGC.GA.G.......G............TGTC..G
consensus     61 *...*.*......********..*....*..*.*******.************....**.

Akkermansia  119 GTAACCTGCCCCCGAGAGCGGGATAGCCCTGGGAAACTGGGATTAATACCGCATAGTATC
Cronobacter  121 .-..A.....TGATG...G......A.TACT......G.TAGC............ACG..
consensus    121 *.**.*****.....***.******.*....******.*....************...**

Akkermansia  179 GAAAGATTAAAGCAGCAA------------TGCGCTTGGGGATGGGCTCGCGGCCTATTA
Cronobacter  180 T.CG..CC....TG.GGGACCTTCGGGCCTCAT..CATCA....T..C.AGATGGG....
consensus    181 .*..**..****..*..................**.....****.**.*.......****

Akkermansia  227 GTTAGTTGGTGAGGTAACGGCTCACCAAGGCGATGACGGGTAGCCGGTCTGAGAGGATGT
Cronobacter  240 .C....A....G..............T......C..TCCC....T..............A
consensus    241 *.****.****.**************.******.**....****.**************.
                                                                Similarity with Akkermansia
Akkermansia  287 CCGGCCACACTGGAACTGAGACACGGTCCAGACACCTACGGGTGGCAGCA
Cronobacter  300 ..A..............................T........A....... 71.43%
consensus    301 **.******************************.********.*******
```

B

```
V34
Akkermansia      1 CCTACGGGTGGCAGCAGTCGAGAATCATTCACAATGGGGGAAACCCTGATGGTGCGACGC
Cronobacter      1 ....T...GT..................................................
Observed_OTU     1 ..................G.G....TT.GG..........C.........CCA..C.T..
consensus        1 ****.***..********.*.****..*..**********.*********...**.*.**

Akkermansia     61 CGCGTGGGGGAATGAAGGTCTTCGGATTGTAAACCCCTGTCATGTGGGAGCAAATTAAAA
Cronobacter     61 ............................................................
Observed_OTU    61 ......CA..-.................................................
consensus       61 ******..**.*************************************************

Akkermansia    121 AGATAGTACCACAAGAGGAAGAGACGGCTAACTCTGTGCCAGCAGCCGCGGTAATACAGA
Cronobacter    121 ............................................................
Observed_OTU   120 ............................................................
consensus      121 ************************************************************

Akkermansia    181 GGTCTCAAGCGTTGTTCGGAATCACTGGGCGTAAAGCGTGCGTAGGCTGTTTCGTAAGTC
Cronobacter    181 ............................................................
Observed_OTU   180 ............................................................
consensus      181 ************************************************************

Akkermansia    241 GTGTGTGAAAGGCGCGGGCTCAACCCGCGGACGGCACATGATACTGCGAGACTAGAGTAA
Cronobacter    241 ............................................................
Observed_OTU   240 ............................................................
consensus      241 ************************************************************

Akkermansia    301 TGGAGGGGGAACCGGAATTCTCGGTGTAGCAGTGAAATGCGTAGATATCGAGAGGAACAC
Cronobacter    301 ............................................................
Observed_OTU   300 ............................................................
consensus      301 ************************************************************

Akkermansia    361 TCGTGGCGAAGGCGGGTTCCTGGACATTAACTGACGCTGAGGCACGAAGGCCAGGGGAGC
Cronobacter    361 ............................................................
Observed_OTU   360 ............................................................
consensus      361 ************************************************************
                                             Similarity with Akkermansia
Akkermansia    421 GAAAGGATTAGATACCCCTGTAGTC
Cronobacter    421 .................TC...... 99.32%
Observed_OTU   420 .................T....... 96.41%
consensus      421 *****************..******
```
