# Supplementary material for: Benchmark of 16S rRNA gene amplicon sequencing using Japanese gut microbiome data from the V1–V2 and V3–V4 primer sets
Source: BMC Genomics. 2021 Jul 10;22:527. doi: 10.1186/s12864-021-07746-4 (PMC8272389; doi:10.1186/s12864-021-07746-4)
Supplement: Supplementary file 2 — Additional file 2: Table 1S. Distribution of the selected participants. Table 2S. Percentage of classified OTUs/ASVs at each classification level. Table 3S. OTUs/ASVs numbers and the percentage of filtered-out sequences. Table 4S. Lists of bacteria that showed statistical differences at the phylum level between V12 and V34. Table 5S. Lists of bacteria that showed statistical differences at the genus level between V12 and V34. Table 6S. Average compositions of the genera Bacteroides and Faecalibacterium. [file 12864_2021_7746_MOESM2_ESM.pdf]

Benchmark of 16S rRNA gene amplicon sequencing using Japanese gut microbiome data from the V1–V2 and V3–V4 primer sets

Shoichiro Kameoka[a,e], Daisuke Motooka[a,b,d], Satoshi Watanabe[e], Ryuichi Kubo[e], Nicolas Jung[a], Yuki Midorikawa[e], Natsuko O. Shinozaki[e], Yu Sawai[e], Aya K. Takeda[e], and Shota Nakamura[a,b,c,d]#

[a]Department of Infection Metagenomics, Genome Information Research Center, Research Institute for Microbial Diseases, Osaka University, Suita, Osaka, Japan

[b]Next-Generation Sequencing Core Facility, Genome Information Research Center, Research Institute for Microbial Diseases, Osaka University, Suita, Osaka, Japan

[c]Laboratory of Pathogen Detection and Identification, International Research Center for Infectious Diseases, Research Institute for Microbial Diseases, Osaka University, Suita, Osaka, Japan

[d]Integrated Frontier Research for Medical Science Division, Institute for Open and Transdisciplinary Research Initiatives, Osaka University, Suita, Osaka, Japan

[e]Cykinso, Inc. Shibuya, Tokyo, Japan

#Address correspondence to Shota Nakamura, nshota@gen-info.osaka-u.ac.jp

Supplementary tables

Table 1S Distribution of the selected participants.

Table 2S Percentage of classified OTUs/ASVs at each classification level.

Table 3S OTUs/ASVs numbers and the percentage of filtered-out sequences.

Table 4S Lists of bacteria that showed statistical differences at the phylum level between V12 and V34.

Table 5S Lists of bacteria that showed statistical differences at the genus level between V12 and V34.

Table 6S Average compositions of the genera *Bacteroides* and *Faecalibacterium*.

Table 1S Distribution of the selected participants

Age group	Male	Female	Total
0	3	2	5
10	0	0	0
20	7	32	39
30	18	46	64
40	13	30	43
50	13	13	26
60	4	2	6
70	1	1	2
80	1	2	3
90	0	2	2
NI*			2
Sum	60	130	192

*No information about gender and age

Table 2S Percentage of classified OTUs/ASVs at each classification level

	Kingdom	Phylum	Class	Order	Family	Genus
V12qI	99.99	99.46	99.12	98.7	93.37	69.92
V34qI	99.53	97.76	96.51	95.38	86.85	64.49
V12qII	100	100	99.83	99.73	92.21	63.04
V34qII	100	99	98.67	98.53	91.15	66.51

Table 3S OTUs/ASVs numbers and the percentage of filtered-out sequences

		OTUs/ASVs numbers				Percentage of filtered-out sequences	
	#Taxon	V12qI	V34qI	V12qII	V34qII	(V12qI - V12qII) / V12qI	(V34qI - V34qII) / V34qI
Classified OTUs/ASVs	k__Bacteria;p__Firmicutes	50016	40943	8516	2750	44.3%	44.8%
	k__Bacteria;p__Bacteroidetes	30457	27660	1615	1249	30.8%	31.0%
	k__Bacteria;p__Proteobacteria	9053	9228	686	491	8.9%	10.3%
	k__Bacteria;p__Actinobacteria	2323	3244	344	173	2.1%	3.6%
	Other phyla	1259	2192	184	163	1.1%	2.4%
		93108	83267	11345	4826	87.3%	92.1%
Unclassified OTUs/ASVs	k__Bacteria;__	491	1406	18	58	0.5%	1.6%
	k__Bacteria;p__	0	0	0	5	0.0%	0.0%
	k__Archaea;__	1	102	0	0	0.0%	0.1%
	Unclassified;__	11	403	0	2	0.0%	0.5%
		503	1911	18	65	0.5%	2.2%
	Total OTUs/ASVs numbers	93611	85178	11363	4891	87.9%	94.3%

Table 4S Lists of bacteria that showed statistical differences at the phylum level between V12 and V34

A

QIIME1		Average composition	
#Taxon	*p*	V12qI	V34qI
k__Bacteria;p__Actinobacteria	2.37E-06	0.026998248	0.043451603
k__Bacteria;p__Verrucomicrobia	2.64E-06	0.002192311	0.020702261
k__Bacteria;Other	2.05E-36	1.20E-04	4.57E-04
Unclassified;Other	1.89E-54	ND	9.75E-05
k__Archaea;Other	2.33E-20	ND	1.85E-05
k__Archaea;p__Euryarchaeota	9.86E-08	ND	5.34E-05

B

QIIME2		Average composition	
#Taxon	*p*	V12qII	V34qII
k__Bacteria;p__Actinobacteria	0.002737425	0.026485609	0.035276621
k__Bacteria;p__Verrucomicrobia	0.005489132	0.002307882	0.019658722
k__Bacteria;Other	2.41E-05	4.16E-04	5.31E-05
k__Archaea;p__Euryarchaeota	0.001383525	0	5.29E-05

ND < 1.00E-06

Table 5S Lists of bacteria that showed statistical differences at the genus level between V12 and V34

A QIIME1: p__Actinobacteria

#Taxon	*p*	Average composition V12qI	V34qI
c__Actinobacteria;o__Bifidobacteriales;f__Bifidobacteriaceae;g__Bifidobacterium	3.10E-04	0.020693978	0.033122488
c__Coriobacteriia;o__Coriobacteriales;f__Coriobacteriaceae;g__Collinsella	0.00473162	0.004398564	0.007998174
c__Actinobacteria;o__Actinomycetales;f__Actinomycetaceae;g__Actinomyces	4.57E-07	1.59E-04	8.90E-05
c__Coriobacteriia;o__Coriobacteriales;f__Coriobacteriaceae;Other	6.23E-11	1.49E-05	7.82E-05
c__Actinobacteria;o__Bifidobacteriales;f__Bifidobacteriaceae;Other	6.07E-11	ND	2.12E-05
c__Coriobacteriia;o__Coriobacteriales;f__Coriobacteriaceae;g__Coriobacterium	1.76E-09	ND	2.53E-05
c__Actinobacteria;Other;Other;Other	1.29E-10	ND	1.16E-05

B QIIME2: p__Actinobacteria

#Taxon	*p*	Average composition V12qII	V34qII
c__Actinobacteria;o__Bifidobacteriales;f__Bifidobacteriaceae;g__Bifidobacterium	0.006644877	0.020746356	0.02858952
c__Actinobacteria;o__Actinomycetales;f__Actinomycetaceae;g__Actinomyces	2.88E-07	7.03E-05	3.16E-05

C QIIME1: p__Verrucomicrobia

#Taxon	*p*	Average composition V12qI	V34qI
c__Verrucomicrobiae;o__Verrucomicrobiales;f__Verrucomicrobiaceae;g__Akkermansia	9.79E-06	0.002174293	0.02047823
Other;Other;Other;Other	4.39E-06	ND	1.02E-05
c__Verrucomicrobiae;o__Verrucomicrobiales;f__Verrucomicrobiaceae;Other	1.19E-14	0	4.35E-05
c__Verrucomicrobiae;o__Verrucomicrobiales;f__Verrucomicrobiaceae;g__Haloferula	2.48E-07	0	ND

D QIIME2: p__Verrucomicrobia

#Taxon	*p*	Average composition V12qII	V34qII
c__Verrucomicrobiae;o__Verrucomicrobiales;f__Verrucomicrobiaceae;g__Akkermansia	0.00891657	0.002289121	0.019467942

ND < 1.00E-06

Table 6S Average compositions of the genera Bacteroides and Faecalibacterium

A

QIIME1		Average composition	
#Taxon	*p*	V12qI	V34qI
g__Bacteroides	0.618542111	0.31800466	0.324943359
g__Faecalibacterium	0.67398998	0.065942334	0.062947882

B

QIIME2		Average composition	
#Taxon	*p*	V12qII	V34qII
g__Bacteroides	0.790085397	0.338854664	0.336794093
g__Faecalibacterium	0.54280411	0.065723894	0.06267233
